# Circulating miRNA expression in extracellular vesicles is associated with specific injuries after multiple trauma and surgical invasiveness

**DOI:** 10.3389/fimmu.2023.1273612

**Published:** 2023-10-23

**Authors:** Rald Victor Maria Groven, Johannes Greven, Ümit Mert, Klemens Horst, Qun Zhao, Taco Johan Blokhuis, Markus Huber-Lang, Frank Hildebrand, Martijn van Griensven

**Affiliations:** ^1^Department of Cell Biology-Inspired Tissue Engineering, MERLN Institute for Technology-Inspired Regenerative Medicine, Maastricht University, Maastricht, Netherlands; ^2^Division of Trauma Surgery, Department of Surgery, Maastricht University Medical Center+, Maastricht, Netherlands; ^3^Experimental Orthopaedics and Trauma Surgery, Department of Orthopaedics, Trauma and Reconstructive Surgery, University Hospital Rheinisch-Westfälische Technische Hochschule (RWTH) Aachen, Aachen, Germany; ^4^Department of Orthopaedics, Trauma and Reconstructive Surgery, University Hospital Rheinisch-Westfälische Technische Hochschule (RWTH) Aachen, Aachen, Germany; ^5^Institute of Clinical and Experimental Trauma Immunology, University Hospital Ulm, Ulm, Germany

**Keywords:** microRNAs, multiple trauma, extracellular vesicles, early total care, damage control orthopaedics

## Abstract

**Introduction:**

Two trauma treatment principles are Early Total Care (ETC), and Damage Control Orthopedics (DCO). Cellular mechanisms that underlie the connection between treatment type, its systemic effects, and tissue regeneration are not fully known. Therefore, this study aimed to: 1) profile microRNA (miRNA) expression in plasma derived Extracellular Vesicles (EVs) from a porcine multiple trauma model at different timepoints, comparing two surgical treatments; and 2) determine and validate the miRNA’s messengerRNA (mRNA) targets.

**Methods:**

The porcine multiple trauma model consisted of blunt chest trauma, liver laceration, bilateral femur fractures, and controlled haemorrhagic shock. Two treatment groups were defined, ETC (n=8), and DCO (n=8). Animals were monitored under Intensive Care Unit-standards, blood was sampled at 1.5, 2.5, 24, and 72 hours after trauma, and EVs were harvested from plasma. MiRNAs were analysed using quantitative Polymerase Chain Reaction arrays. MRNA targets were identified *in silico* and validated *in vivo* in lung and liver tissue.

**Results:**

The arrays showed distinct treatment specific miRNA expression patterns throughout all timepoints, and miRNAs related to the multiple trauma and its individual injuries. EV-packed miRNA expression in the ETC group was more pro-inflammatory, indicating potentially decreased tissue regenerative capacities in the acute post-traumatic phase. *In silico* target prediction revealed several overlapping mRNA targets among the identified miRNAs, related to inflammation, (pulmonary) fibrosis, and Wnt-signalling. These were, among others, A Disintegrin and Metalloproteinase domain-containing protein 10, Collagen Type 1 Alpha 1 Chain, Catenin Beta Interacting Protein 1, and Signal Transducers and Activators of Transcription 3. Validation of these mRNA targets in the lung showed significant, treatment specific deregulations which matched the expression of their upstream miRNAs. No significant mRNA deregulations were observed in the liver.

**Discussion:**

This study showed treatment specific, EV-packed miRNA expression patterns after trauma that correlated with mRNA expressions in the lungs, target organs over distance. A systemic response to the increased surgical trauma in the ETC group was identified, with various miRNAs associated with injuries from the trauma model, and involved in (systemic) inflammation, tissue regeneration. EV-transported miRNAs demonstrated a clear role in multiple trauma, warranting further research into tissue-tissue talk and therapeutic applications of EVs after trauma.

## 1 Introduction

Multiple trauma results in a high mortality rate and often invalidating consequences, in part because the sustained injuries can elicit exacerbated immune responses throughout the body, causing conditions such as Systemic Inflammatory Response Syndrome (SIRS) and Compensatory Anti-inflammatory Response Syndrome (CARS) (1). Re-establishing an immunological balance as early as possible after multiple trauma is therefore of vital importance, since this will aid in stabilising the patient and speeding up their recovery. The so called “second hit” inflicted by the surgical trauma potentially aggravates conditions such as SIRS and CARS, warranting for a careful choice between Damage Control Orthopaedics (DCO) and Early Total Care (ETC) in the initial post-traumatic phase (2).

The main difference between DCO and ETC is the invasiveness of the initial fracture treatment. The temporary fixation of long bone fractures in DCO is used to ensure a physiological steady state, prior to converting to more invasive surgical fracture fixation procedures (3). However, primary temporary fixation requires more follow up surgeries which prolongs the inflammatory response and immobilization time. The main aim of ETC is to definitively fixate all long bone fractures within primary fracture surgery, shortening the overall inflammatory response and allowing for more swift recovery (4).

Several studies have identified specific indications for either DCO or ETC, but their exact systemic cellular mechanisms that underlie their differential effects on tissue regeneration after multiple trauma are not yet fully understood (4, 5). Cellular signalling, communication, and functioning are important to adequately respond to traumatic tissue damage, inflammation, and enable for tissues to regenerate. These processes are, in part, facilitated by Extracellular Vesicles (EVs), which are important transporters of cellular mediators. EVs have been increasingly investigated in trauma due to their involvement in the underlying pathophysiology, tissue regeneration, and their ability to enable organ crosstalk (6). The latter is of particular interest in a rather heterogenic condition such as multiple trauma (7). Furthermore, research has shown that multiple trauma enhances the amount of the circulating EVs and influences their composition (8).

Upon stress, cells can release EVs to deliver various compounds, such as lipids, proteins, and microRNAs (miRNAs) at target cells or organs via the systemic circulation (9). MiRNAs are short, non-coding RNA molecules that post-transcriptionally regulate protein expression by blocking messengerRNA (mRNA) translation, or activating the transcription of specific genes by enhancing promoter activity (10). However, the involvement of miRNAs in the field of trauma surgery has predominantly been addressed in the context of local tissue responses, such as bone regeneration (11). Characterization of post-traumatic, systemic miRNA-based cellular communication, e.g. as EV-packed miRNAs, is missing. In particular, the effects of the applied surgical treatment, ETC versus DCO, on the expression of EV-packed miRNAs is still unknown. Transport of EVs to injured organs may facilitate cellular communication over distance in multiple trauma, in part through miRNAs (12).

Taking the above-described characteristics of both EVs and miRNAs into account, we hypothesise that EV-packed miRNAs may be involved in various post-traumatic processes such as immunoregulation and tissue regeneration. A first step in better understanding the role of miRNAs in this complex systemic communication mechanism would therefore be to profile the miRNA fingerprint of circulating EVs after multiple trauma at various timepoints, comparing different surgical treatment strategies.

Therefore, we defined two aims: 1) to profile the expression of miRNAs in plasma-derived EVs from a porcine multiple trauma model at different timepoints after trauma, comparing two different surgical treatment modalities; and 2) to determine and validate the miRNA’s mRNA targets.

## 2 Methods

### 2.1 Animal care and experimental groups

The data presented in this paper were collected in the context of a larger study in accordance with the 3R principles as set out by the national centre for the replacement, refinement, and reduction of animals in research (13). All sections of this manuscript adhere to the ARRIVE Guidelines for reporting animal research (14). The study was approved by the German governmental office of animal care and use LANUV (Landesamt für Natur, Umwelt und Verbraucherschutz Nordrhein-Westfalen, Recklinghausen, Germany) under the permit number 81.02.04.2020.A215.

All animals were clinically examined by a veterinarian after arriving at the hosting facility. They were subsequently housed for seven days prior to the experiment to acclimatise. For the experiments, a total of 22 male German Landrace pigs (*Sus scrofa*) of 35 ± 5 kg of body weight and 3 months of age were used. The animals were divided in three experimental groups: sham (n=6), ETC (n=8), and DCO (n=8). The sham group received identical instrumentation, anaesthesia, mechanical ventilation, nutrition, and, if required, vasopressive therapy. After multiple trauma induction, either intramedullary nailing (ETC) (T2 System, Stryker GmbH & Co. KG, Duisburg, Germany) or external fixation (DCO) (Radiolucent Fixator, Orthofix, Texas, USA) of both femoral fractures was performed. Parental nutrition (Aminoven, Fresenius Kabi, Germany) was provided under close monitoring of the fluid balance and vasopressive therapy was provided as required.

### 2.2 Instrumentation and anaesthesia

The model has been previously described in detail (15). Briefly, after 12 hours (h) of fasting with water *ad libitum*, azaperone (Stresnil™, Janssen-Cilag GmbH, Neuss, Germany) and ketamine (Ketanest, Pfizer, New York, USA) were injected intramuscularly as premedication. Intravenous propofol (Fresenius SE & Co. KGaA, Homburg, Germany) was used to induce anaesthesia, after which the animals were orotracheally intubated (7.5 ch; Hi-Lo Lanz™, Tyco Healthcare, Hampshire, England). For the administration of fluids, anaesthesia, and continuous monitoring of central venous pressure, a catheter (Four-Lumen Catheter, 8.5 Fr., Arrow Catheter, Teleflex Medical GmbH, Fellbach, Germany) was placed in the external jugular vein. A three-lumen haemodialysis catheter (12.0 Fr., Arrow Catheter, Teleflex Medical GmbH, Fellbach, Germany) and an arterial line (Vygon GmbH & Co. KG, Aachen, Germany) were placed in the right femoral vein and artery to monitor arterial blood pressure and induce haemorrhagic shock. All animals received a suprapubic catheter (12.0 Fr, Cystofix^®^, B. Braun AG, Melsungen, Germany). For the duration of the study, general anaesthesia was maintained using intravenous propofol, sufentanyl, and midazolam (Panpharma GmbH, Trittau, Germany). Proper fluid intake was guaranteed by continuous crystalloid infusion (Sterofundin ISO^®^; 2 ml/kgBW/h) (B. Braun AG, Melsungen, Germany). Mechanical, porcine adapted volume-controlled ventilation was applied (8-12 ml/kgBW); Positive End-Expiratory Pressure (PEEP) 8 mmHg (plateau pressure < 28 mmHg) adjusted by capnometry aiming for a pCO_2_ of 35–45 mmHg (Draeger Evita 4, Draeger Safety AG & Co. KGaA, Lübeck, Germany). The following vital parameters were monitored and, in part, depicted in **Table 1**: body temperature, blood pressure, heart- and breathing-rate, electrocardiogram recordings, electrocardiogram-synchronised pulse oximetry. Furthermore, blood C-reactive protein concentration, leukocyte count, and lactate were determined at pre-determined timepoints (**Table 1**).

**Table 1:** Overview of the following clinical parameters: mean arterial pressure (MAP), heart rate, noradrenaline requirement, C-reactive protein (CRP) concentration, leukocyte count, and lactate.

	1.5 hours	2.5 hours	24 hours	72 hours
MAP (mmHg; IQR)				
*ETC* *DCO*	40; 29 39; 1	65; 8 77; 16	64; 6 64; 10	65; 20 69; 12
Heart rate (BPM; IQR)				
*ETC* *DCO*	99; 63 74; 20	76; 55 78; 30	55; 52 72; 33	72; 11 65; 31
Noradrenalin (ml/h; IQR)				
*ETC* *DCO*	0.1; 0.4 0.1; 0.1	0.1; 0 0.1; 0	2.8; 2.9 0.1; 0	1.8; 2.6 0.1; 0
CRP (mg/l; IQR)				
*ETC* *DCO*	2.0; 2.0 2.0; 3.0	1.0; 3.0 3.0; 3.5	5.0; 4.0 6.5; 5.8	9.5; 4.8 5.0; 3.3
Leukocytes (10^3^/µL; IQR)				
*ETC* *DCO*	18.2; 5.7 18.1; 11.7	19.0; 5.5 17.3; 9.5	18.8; 16.4 13.7; 15.7	8.7; 8.2 11.2; 7.8
Lactate (mmol/l; IQR)				
*ETC* *DCO*	2.2; 2.2 1.7; 1.0	1.6; 1.4 0.9; 1.0	0.5; 0.4 0.4; 0.3	0.4; 0.1 0.4; 0.2

Values are represented as median accompanied by the interquartile range (IQR).

### 2.3 Trauma induction

Prior to multiple trauma induction, baseline conditions were required to be stable for at least 120 min after instrumentation. The fraction of inspired oxygen (FiO_2_) was set at 0.21 throughout instrumentation, as well as during multiple trauma induction and the subsequent shock phase to mimic ambient air. During the 90 min shock phase following multiple trauma induction, body temperature was not controlled to mimic the real life, out of hospital environment and fluid administration was set to 0.1 ml/kgBW/h to keep infusion lines open for later usage. The surgical treatment groups received the standardised multiple trauma (ISS=27) as described below.

A bolt gun machine (Blitz-Kerner, turbocut Jopp GmbH, Bad Neustadt an der Saale, Germany) with cattle-killing cartridges (9 × 17; DynamitNobel AG, Troisdorf, Germany) was used to induce blunt chest trauma by firing the bolt gun machine onto a pair of steel and lead panels (steel 8 mm and lead 10 mm) that were placed on the right dorsal, lower chest. Bilateral femur fractures were induced using a custom-made punch and the bolt gun machine, positioned on the mid-third of the femora.

To mimic liver laceration, a crosswise incision (4.5 x 4.5 cm) half of the tissues’ depth was made in the upper left liver lobe. Following 30 s of uncontrolled bleeding, liver packing was performed with five sterile packs of 10 x 10 cm gauze. Haemorrhagic shock was performed by withdrawing maximally 45% of the total blood volume, until a Mean Arterial Pressure (MAP) of 40 ± 5 mmHg was achieved. The combined multiple trauma was left untreated for 90 min to mimic a real life shock phase. If sham animals required vasopressive therapy, this was also only provided after 90 min.

After the shock phase, animals were resuscitated in accordance with existing trauma guidelines (ATLS^®^, AWMF-S3 guideline on Treatment of Patients with Severe and Multiple Injuries^®^) by adjusting the FiO_2_ to base values for mechanical ventilation, re-infusion of the withdrawn blood (Citrate Phosphate Dextrose Adenine DONOpacks, Lmb Technologie GmbH, Oberding, Germany) as well as fluids administration (Sterofundin ISO^®^; 2 ml/kgBW/h). A forced-air warming system was used to achieve and/or maintain normothermia for the animals (*sus scrofa*: German landrace) (38.7–39.8 °C). Prior to surgery and every 24 h after multiple trauma induction, 2 g of ceftriaxone (Fresenius SE & Co. KGaA, Homburg, Germany) were administered via intravenous infusion. Animals were turned every four to six hours to support the respiratory mechanics in the mechanically ventilated animals.

### 2.4 Sample collection and EV isolation

Per animal of each experimental group, 1 ml of plasma was collected at the following timepoints: 1.5, 2.5, 24, and 72 h. For the isolation of EVs, samples were subjected to the following standard operating procedure, isolating EVs from 30 to 1000 nm. Plasma samples were sequentially centrifuged at 300 x g for 10 min, 2000 x g for 15 min, and 5000 x g for 15 min at 4°C. Cells, cell debris, and apoptotic bodies were eliminated respectively. The EVs were then pelleted by further centrifugation at 20000 x g for 90 min at 4°C (Avanti J-26XP, Beckman Coulter BV, Woerden, the Netherlands). The acquired precipitate was gathered and suspended in 100 µl Phosphate Buffered Saline (Sigma-Aldrich, St Louis, USA) (52, 53). The collected EVs were then stored at − 80°C.

### 2.5 RNA isolation and cDNA synthesis

RNA extraction was performed by chloroform phenol extraction, using 1 ml of Trizol reagent (Thermo Fisher Scientific, Waltham, USA) per 100 µl EV containing suspension. Furthermore, GlycoBlue co-precipitant (Thermo Fisher Scientific, Waltham, USA) was used according to the manufacturer’s instructions. The amount and purity of the RNA were determined by spectrophotometry using a CLARIOstar Plate Reader and LVis Plate Adapter (Isogen Life Science, De Meern, The Netherlands), after which the RNA was stored at -80˚C. Per animal, RNA was then used for the transcription of miRNA to cDNA using the miRCURY LNA RT kit (Qiagen, Venlo, The Netherlands) according to manufacturer’s instructions.

### 2.6 miRNA qPCR arrays

For this study, miRCURY LNA miRNA Serum/Plasma Focus PCR panels (YAHS-106YD-8, Qiagen, Venlo, The Netherlands) were used. This panel kit consists of 179 primers for unique miRNAs, excluding several PCR and reverse transcription controls, housekeeper genes, and interplate calibrators. The expression of miRNAs was determined in the pooled sample per respective treatment group and for each specific timepoint. Gene expression was normalised according to the global Cq method. Sham samples of the respective timepoints were used as control. MiRNA gene expression levels were determined via qPCR by the cycle number (Cq), using the CFX96 Real-Time PCR system (Bio-Rad GmbH, Munich, Germany). To assess the quality of the qPCR array reactions, melting curve analyses were performed in combination with a chosen cut off Cq value of 35, above which miRNAs were not considered for further analysis due to minute or absent presence. Fold regulation (FR) was calculated using comparative 2^−ΔΔCt^ method, in which a downregulation was represented as the negative inverse of the acquired 2^−ΔΔCt^ value. A deregulation, as represented by a FR of ≥ 2 or ≤ -2, was considered significantly deregulated. For each timepoint, a selection of the 20 most deregulated miRNAs among the two treatment groups was composed, and from that, miRNAs that were deregulated at minimally two timepoints were selected for bioinformatic analysis and mRNA target validations.

### 2.7 Bioinformatic analyses of miRNA-mRNA interactions

Possible miRNA-mRNA interactions were estimated using the analytical platform tools4miRs as well as an extensive literature review. Five different mRNA target prediction algorithms were applied: miRanda, miRmap, RNA22, RNAhybrid, and TargetSpy. A main focus was put on genes that were involved in multiple trauma, SIRS, CARS, MODS, sepsis, and bone regeneration. A miRNA-mRNA interaction was included if it resulted as a true hit in minimally three algorithms.

### 2.8 mRNA target validation

To examine the systemic effects of the deregulated miRNA *in vivo*, a selection of mRNAs of interest was analysed in lung and liver samples by means of qPCR based on the *in silico* target prediction and literature review. The following genes were included for qPCR: A Disintegrin and Metalloproteinase domain-containing protein 10 (ADAM10), Collagen Type 1 Alpha 1 Chain (COL1A1), Catenin Beta Interacting Protein 1 (CTNNBIP1), and Signal Transducers and Activators of Transcription 3 (STAT3). The amplification primers are listed in **Supplemental Table 1**. Controls included positive and negative PCR controls, as well as reverse transcription controls.

MRNA expression levels were determined via qPCR by the cycle number (Cq), using a CFX96 Real-Time PCR system (Bio-Rad GmbH, Munich, Germany). To assess the quality of the individual qPCR reactions, melting curve analyses were performed in combination with a chosen cut off Cq value of 35, above which mRNAs were not considered expressed. Data were processed using comparative 2^−ΔΔCt^ method, using GAPDH as a housekeeper gene. A FR of ≥ 2 or ≤ -2 was considered significantly deregulated.

### 2.9 Statistical analyses

Analyses were performed with GraphPad Prism version 9.5.0 (GraphPad Software, San Diego, USA). Shapiro-Wilks test was performed to assess normality. Data are represented as mean or median, accompanied by standard error of the mean or interquartile range as appropriate. Significance was determined using one-way ANOVA with Holm-Šidák’s multiple comparison test. A p ≤ 0.05 was considered statistically significant.

## 3 Results

**Figure 1:**
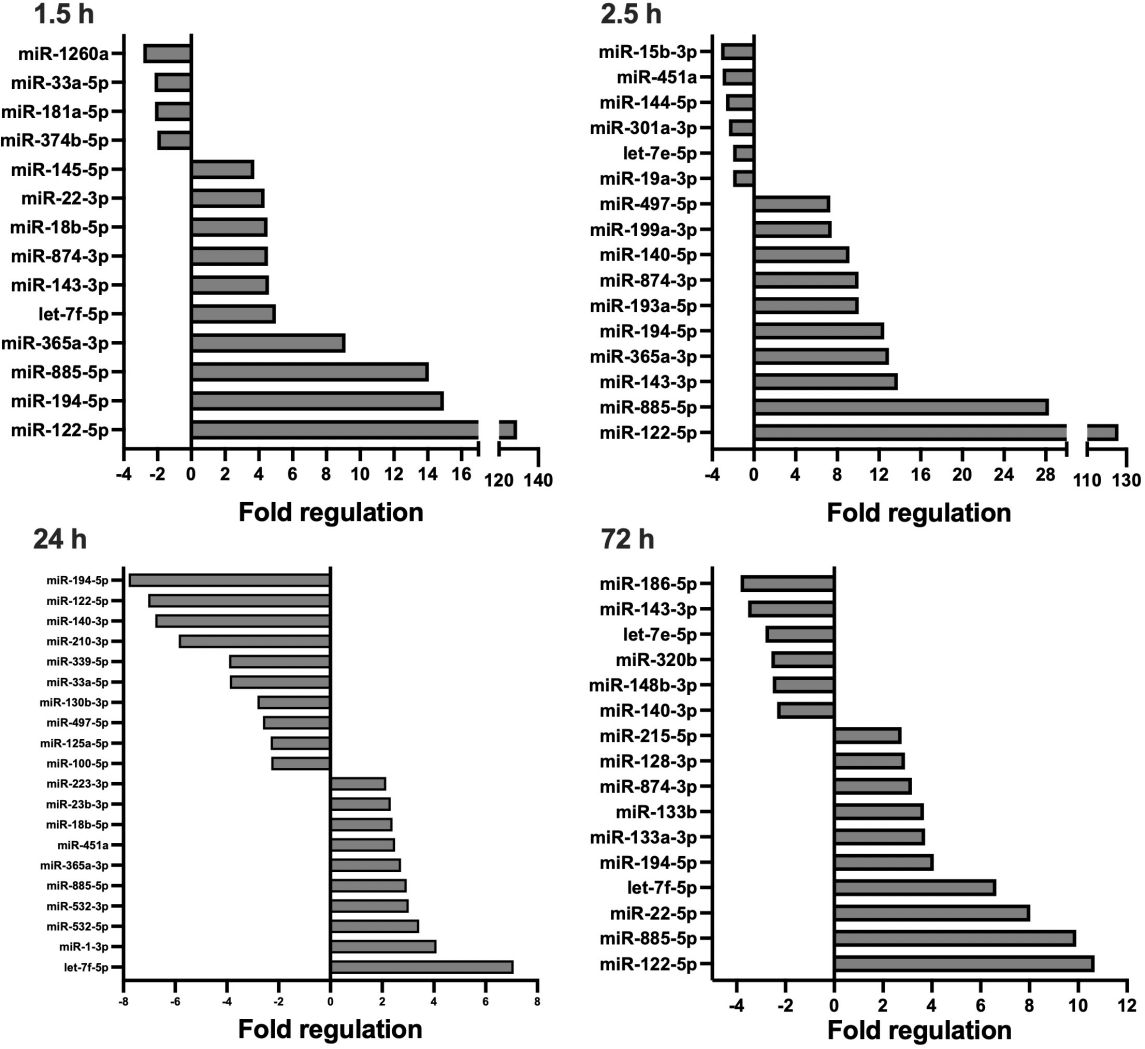
Circulating, extracellular vesicle-packed miRNA expression signature of the ETC group at four timepoints after trauma: 1.5, 2.5, 24, and 72 h.. All depicted miRNAs were significantly deregulated at the respective timepoint. A maximum of 10 up- and downregulated miRNAs are shown. Fold regulation (FR) was calculated using comparative 2^−ΔΔCt^ method, in which a downregulation was represented as the negative inverse of the acquired 2^−ΔΔCt^ value.

**Figure 2:**
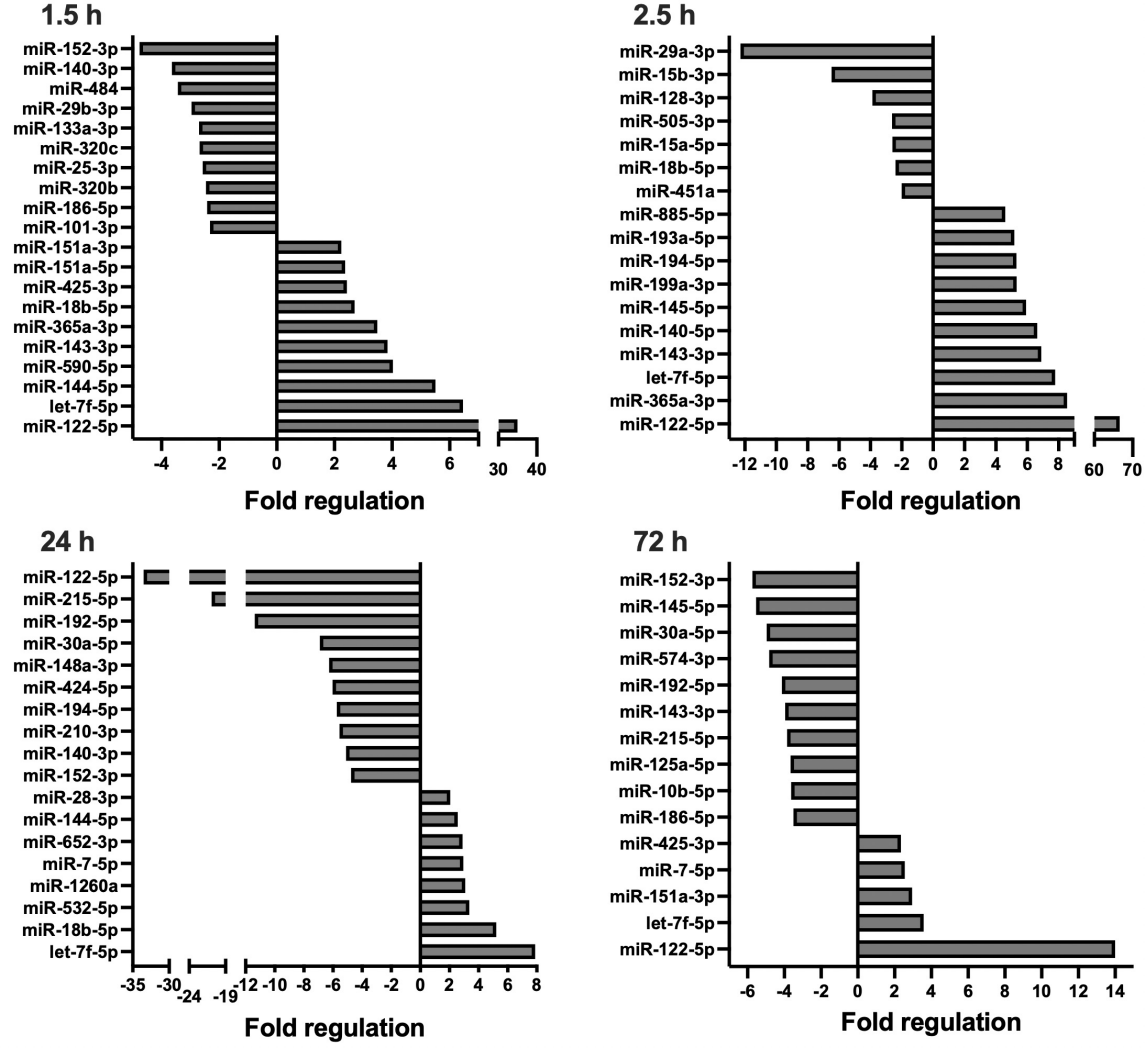
Circulating, extracellular vesicle-packed miRNA expression signature of the DCO group at four timepoints after trauma: 1.5, 2.5, 24, and 72 h.. All depicted miRNAs were significantly deregulated at the respective timepoint. A maximum of 10 up- and downregulated miRNAs are shown. Fold regulation (FR) was calculated using comparative 2^−ΔΔCt^ method, in which a downregulation was represented as the negative inverse of the acquired 2^−ΔΔCt^ value.

Overall, 179 unique miRNAs were examined per timepoint, for each treatment group. In total, 136 miRNAs were deregulated at minimally one timepoint after multiple trauma. **Figures 1**, **2** display significantly deregulated miRNAs, maximally 10 up and downregulated per timepoint, from the ETC and DCO groups respectively. **Table 1** displays the following vital parameters, monitored on the respective timepoints: mean arterial pressure (MAP), heart rate, noradrenaline requirement, and blood C-reactive protein concentration, leukocyte count, and lactate.

In short, compared to the sham control, four and 40 EV-derived miRNAs were respectively down- and upregulated at 1.5 h after multiple trauma in the ETC group. Throughout the acute post-traumatic phase, at 2.5 h after trauma, the number of upregulated miRNAs increased to 51 while six miRNAs were downregulated. At 24 h after multiple trauma, the number of downregulated miRNAs doubled in the ETC group, while the total of upregulated miRNAs decreased from 51 to 12. At 72 h after multiple trauma induction, the overall miRNA expression profile normalised to baseline. Overall, the early circulatory EV-derived miRNA expression signature of the ETC group showed strong deregulations of both anti- and proinflammatory miRNAs, such as miR-29a and miR-192 respectively, as well as miRNAs related to angiogenesis, such as miR-122, and the formation of granulation tissue/fibrosis, such as miR-133a. At 72 h, these expression profiles changed, showing increased expression of miRNAs important for cellular proliferation and differentiation, as well as osteogenesis.

Compared to the sham control, miRNA expression profiles in the DCO group showed significant differences at all timepoints, most prominently at 24 h. For all timepoints, the number of downregulated miRNAs was higher in the DCO group compared to the ETC group. At 1.5 h after multiple trauma, 17 and 13 miRNAs were respectively down- and upregulated. The expression profile changed within the following hour, resulting in 7 down- and 42 upregulated miRNAs at 2.5 h after trauma. This trend reversed itself at 24 h, showing a significant drop in the number of overall upregulated miRNAs to 8, and an increase in the total number of downregulated miRNAs to 27. Lastly, at 72 h after multiple trauma induction, miRNA expression showed strong downregulations, with 35 down- and merely 5 upregulated miRNAs (**Supplemental Table 2**). Collectively, EV-derived miRNA expression in the acute post-traumatic phase was characterised by an increased expression of anti-inflammatory miRNAs, while also showing both up and downregulations for several miRNAs that are involved in cellular proliferation, migration, and cellular senescence. The later timepoints showed downregulations of miRNAs that were, e.g., anti-apoptotic such as miR-29a, while showing upregulations of miRNAs that enhanced cellular proliferation and differentiation in the ETC group, such as miR-885 and miR-29c.

### 3.1 Most deregulated miRNAs

**Figure 3:**
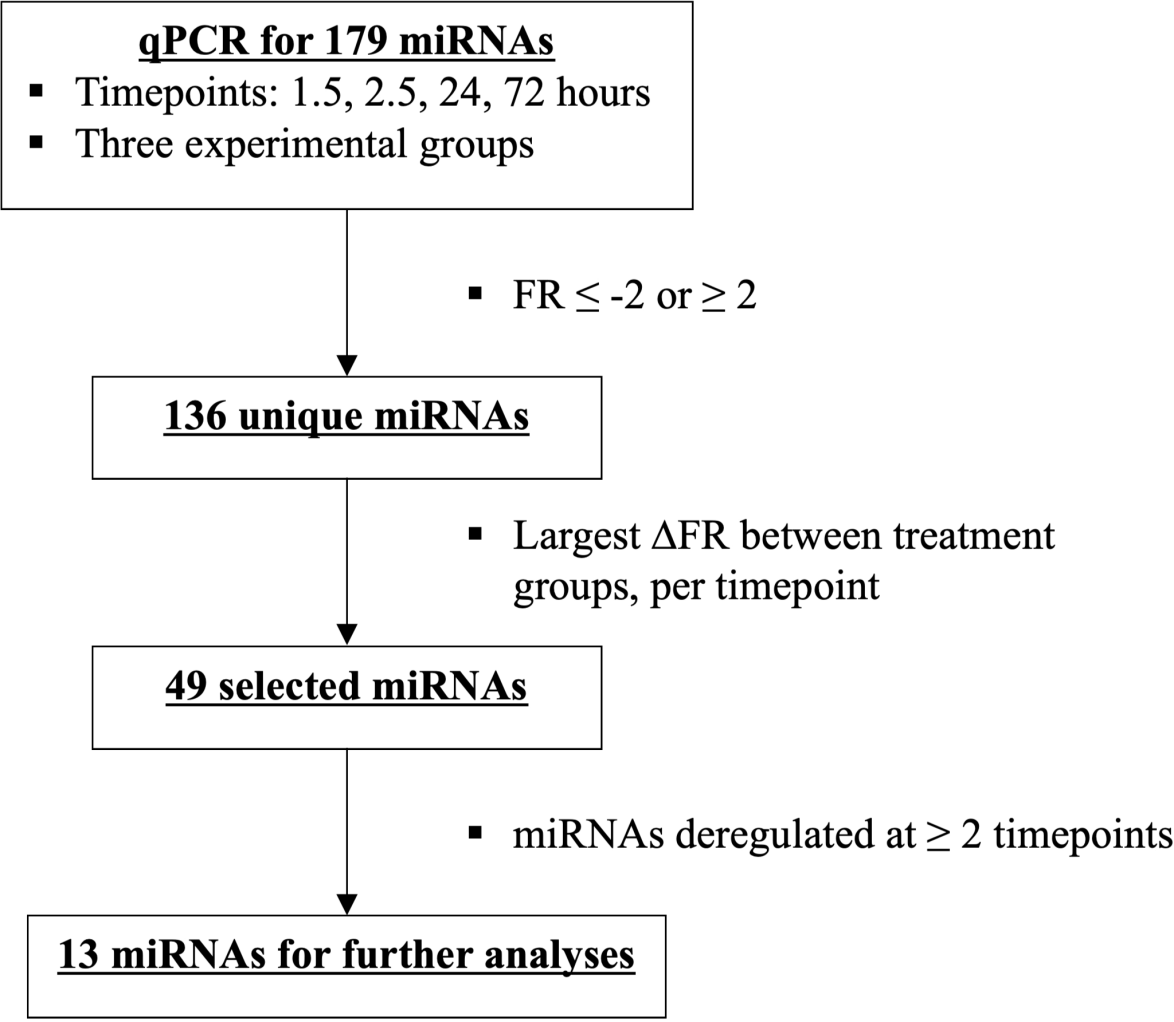
Flowchart depicting miRNA selection of the most deregulated miRNAs among the two treatment groups, ETC vs DCO.

**Figure 4:**
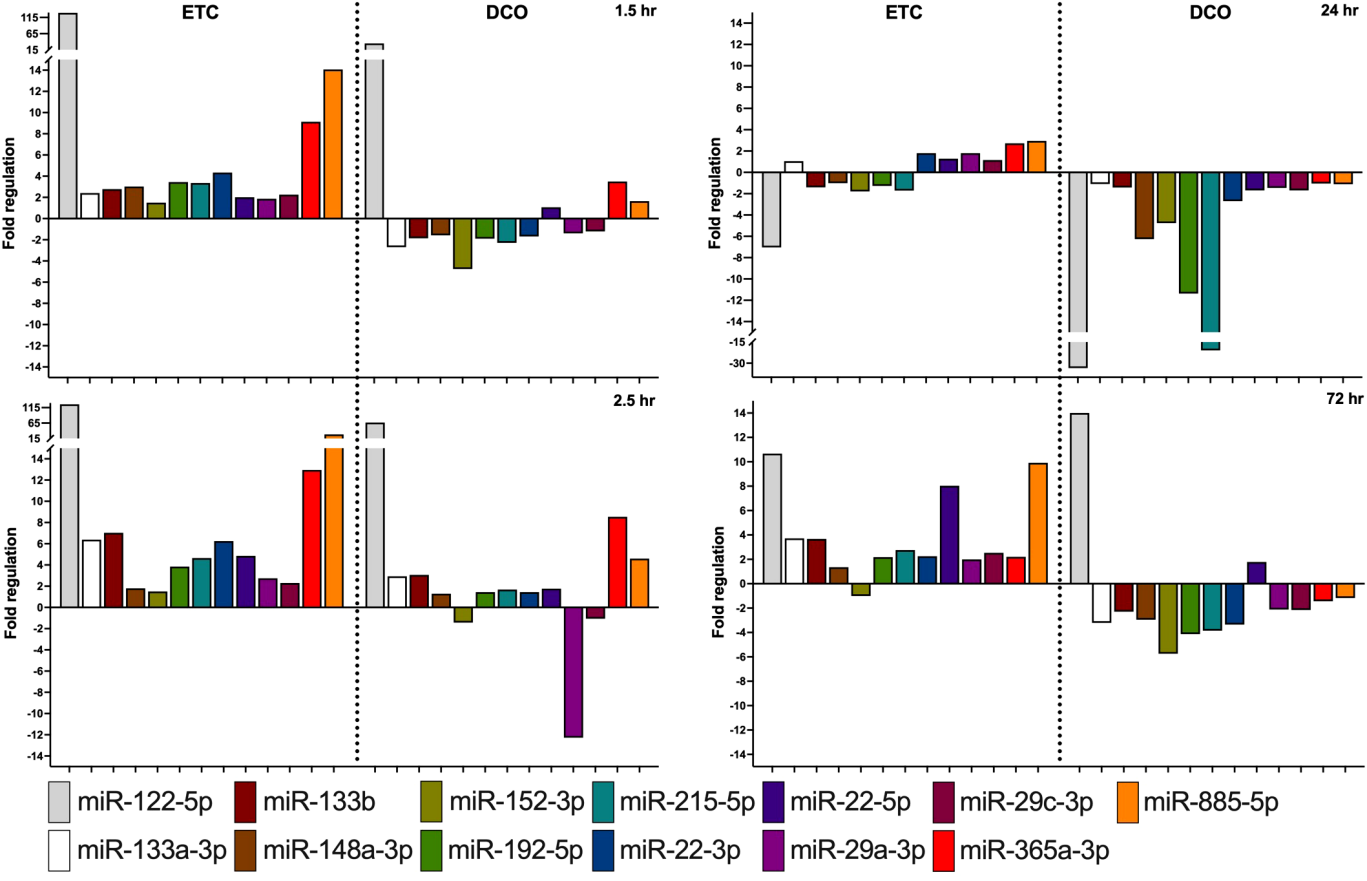
Most significantly deregulated miRNAs between the ETC and DCO treatment groups. All depicted miRNAs were significantly up or downregulated at minimally two timepoints after surgical intervention. Fold regulation (FR) was calculated using comparative 2^−ΔΔCt^ method, in which a downregulation was represented as the negative inverse of the acquired 2^−ΔΔCt^ value.

MiRNAs that exhibited the greatest difference in fold regulation (FR) between the two surgical treatment groups at minimally two timepoints after trauma were selected from the identified 136 unique miRNAs. In total, this provided 13 miRNAs (**Figures 3**, **4**).

The more invasive character of ETC resulted in an overall upregulation of EV-packed miRNAs. In the acute phase until 2.5 h after multiple trauma, most deregulated miRNA expressions were present in the ETC group, with a steady upregulation over time for several specific miRNAs as compared to control. Contrarily, the DCO group showed less deregulation at 1.5 h than ETC, as compared to baseline. At 2.5 h, various miRNAs revealed significant up- and downregulations. For the ETC group, EV-derived miRNA expression started to normalise at 24 h after multiple trauma, whereas the DCO group demonstrated evident downregulations for all of the selected miRNAs. These highly significant downregulations in the DCO group at 24 h after trauma normalised at 72 h. For the ETC group, strong upregulations were present at 72 h for few miRNAs, but the overall expression profile remained comparable to that of 24 h after trauma.

### 3.2 Bioinformatic analyses

Tools4miRs identified 214 targets for the 13 selected miRNAs. Targets were involved in various aspects of the immune responses and tissue regeneration, such as response to hypoxia, chemotaxis, inflammation, apoptosis, cellular migration and differentiation, and fibrosis. Interestingly, several miRNAs and their targets were identified that matched (organ) specific injuries from the multiple trauma model. Examples of these are the hepato-specific miR-122, miRNAs 29a and 133 with key functions in pulmonary fibrosis, and the involvement of miRNAs 133 and 192 in systemic inflammation. The *in silico* target prediction and literature review showed that several mRNA targets overlapped between the identified miRNAs.

### 3.3 qPCR for mRNA targets

**Figure 5:**
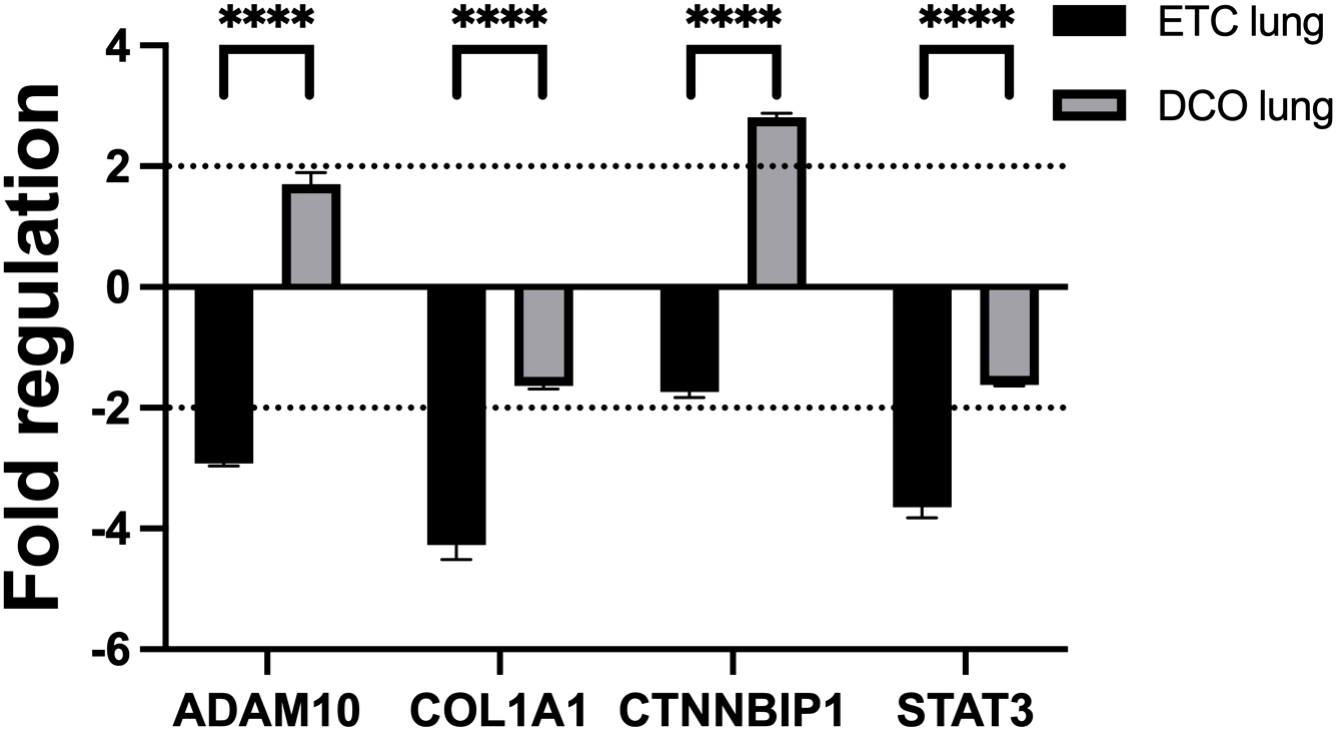
miRNA targeted mRNAs are deregulated in lung tissue, depending on the applied surgical treatment strategy: intramedullary nailing (Early-Total-Care) versus external fixation (Damage-Control-Orthopaedics). Black dotted line displays the threshold of a fold regulation of |≥ 2| above or below which a mRNA was considered significantly deregulated. Significant differences in mRNA expression between the same sample types from different treatment groups are marked as follows: ****p-value<0.0001. Error bars depict standard error of the mean.

Four key mRNA targets of the deregulated miRNAs were analysed by means of qPCR to assess the potential effect of the deregulated miRNAs in target organs. The mRNA targets were selected based on the bioinformatic analyses combined with the overview of the most deregulated, EV-packed miRNAs between the two treatment groups. A total of four miRNA targets, ADAM10, COL1A1, CTNNBIP1, and STAT3, were chosen for validation in lung and liver samples, based on their functions in tissue regenerative processes in these organs, such as in (pulmonary) fibrosis after trauma, regulation of the inflammatory response, Wingless-related integration site signalling pathway (Wnt-signalling), and liver- and alveolar-tissue homeostasis. MiR-122 and 365, both showing significant deregulations over time, were among the upstream targets of ADAM10. Furthermore, miR-133a, miR-29a, and -29c targeted COL1A1, while CTNNBIP1 was the mRNA target of miR-215 and -885. Lastly, STAT3 was regulated by miR-22 and -29a. Lung and liver samples were of interest due to the nature of the multiple trauma, combined with the observed tissue specific miRNA expression profiles. In the lung, the four analysed mRNA targets were significantly deregulated between the treatment groups (all p < 0.0001). Three of the four mRNA targets, ADAM10, COL1A1, and STAT3, were significantly downregulated in lung tissue from the ETC group. One of the four mRNA targets, CTNNBIP1, was significantly upregulated in lung tissue from the DCO group. These mRNA deregulations were consistent with the deregulation of its upstream miRNAs as well as the *in silico* target prediction (**Figure 5**). However, no significant deregulations were observed in the liver.

## 4 Discussion

Multiple trauma is often complicated by severe, complex immune responses both locally at the sites of injury, as well as in the systemic circulation, influencing tissue damage clearance and regenerative processes (1). This study examined the systemic post-traumatic response by profiling the expression of EV-packed miRNAs in a porcine multiple trauma animal model. In this clinically relevant model, two different surgical treatment methods were compared, ETC and DCO, to examine the effect of surgical invasiveness on miRNA-based organ crosstalk after multiple trauma, using EVs as carriers.

Looking at the miRNA expression data, the individual injuries of the multiple trauma model seem to be represented in the systemic EV-packed miRNA expression. MiR-122 for example, the most abundant liver-specific miRNA, was strongly upregulated in both treatment groups in the acute phase after trauma. This miRNA is a specific systemic biomarker for hepatocyte damage upon acute liver injury, modelled in this current porcine multiple trauma model (16). Consistent with these findings, miR-365 was upregulated in both treatment groups. MiR-365 is an upstream inducer of pro-apoptotic protein synthesis and may be involved in programmed cell death after trauma (17). The kinetics of these two miRNAs were similar between the treatment groups, showing significant upregulations in the acute post-traumatic phase followed by decreased expression at 24 h. The greater upregulation of the hepato-specific miR-122 in the ETC group could indicate that more invasive surgery in the acute phase after multiple trauma aggravates hepatocyte damage, potentially through increased systemic inflammation. This is in line with clinical findings in which the stress response to enhanced surgical trauma increases the risk of liver decompensation in patients with chronic liver disease (18). Unfortunately, translational research on the circulating expression of miR-122 and its potential correlations with liver function in patients suffering from multiple trauma without prior liver disease is lacking.

Both miR-365 and miR-122 have also been connected to pulmonary inflammation (16, 19, 20). MiR-365 elicits anti-inflammatory effects by reducing the expression of inflammatory cytokines after IL-17 provocation in asthmatic inflammation during bronchial asthma (19). MiR-122, accounting for 70-80% of the total miRNA expression in the liver, has proven to elicit acute pulmonary inflammation after liver damage, and aggravate pulmonary infections (20, 21). Moreover, increased circulating miR-122 due to acute liver damage has also been associated with mortality in patients suffering from ARDS (16). The fact that both these miRNAs can affect the lungs are in accordance with the findings from this study in which upregulations of circulating, EV-packed, miR-122 and miR-365 were observed, combined with matching downregulations of its mRNA targets in the lungs. Among others, ADAM10 is targeted by miR-122 and miR-365 and was significantly downregulated in lung tissue from the ETC group, matching the stronger upregulations of both miRNAs in this treatment group (**Figures 4**, **5**).

MiR-885 has also shown associations with both lung and liver pathologies, in part by targeting the Wnt-signalling. It was overexpressed at all timepoints in the ETC group, most evidently in the acute phase after trauma. Apart from being a tumour suppressor gene, miR-885 expression has also shown positive correlations with increased serum liver enzymes (22, 23). Furthermore, miR-885 can alleviate bronchial epithelial cell injury (24). Again, the invasiveness of the applied surgical technique influences systemic, EV-packed expression of this miRNA. While being upregulated at all timepoints in the ETC group, it has only shown a comparatively moderate upregulation at 2.5 h after trauma in the DCO group.

Another key component of the multiple trauma model was the induced haemorrhagic shock. Haemorrhagic shock drives immune and organ failure and contributes to among other SIRS, subsequent CARS, and potentially sepsis (1, 2, 25, 26). MiRNAs 133a and 192 are considered potential biomarkers for the development and severity of sepsis and SIRS (27–29). Both miRNAs showed significant deregulations in both treatment groups, being upregulated at almost all timepoints in the ETC group, while revealing significant downregulations in the DCO group, particularly at 24 and 72 h after trauma. These treatment specific deregulations match the increased surgical trauma and subsequent systemic inflammation, keeping their functions in mind. Consistent with these findings was the expression of COL1A1, a mRNA target of miR-133a, in lung tissue from both treatment groups. COL1A1 expression was downregulated in the ETC group, matching the marked upregulations of its upstream miR-133a.

In fact, the targeting of COL1A1 by miR-133a is an important factor in its involvement in pulmonary fibrosis. Overexpression of miR-133a has been reported to be anti-fibrotic and is thereby involved in the physiological response upon lung injury (30). Another miRNA that is involved in the pathogenesis of pulmonary fibrosis is miR-29a. In fact, a miR-29a mimic has been developed to target COL1A1 as a treatment option for pulmonary fibrosis (31). Furthermore, miR-29a reduces alveolar epithelial PANoptosis *in vivo* by which it improves acute lung injury (32). MiR-133a and miR-29a exhibited similar expression kinetics within each treatment group, showing most deregulation in the ETC group, consistently upregulated at all timepoints. This overexpression could thus protect against pulmonary fibrosis and inflammatory cell death upon blunt chest trauma (30–32). The expression of COL1A1 in lung tissue from both treatment groups is consistent with its upstream regulator miR-29a (**Figures 4**, **5**).

MiR-29a also has systemic implications in relation to sepsis by targeting STAT3, a key regulator of the inflammatory response in sepsis (33). Like miR-29a, miR-22 also targets STAT3. Both miRNAs resulted similarly deregulated in both treatment groups, with the significant downregulation in pulmonary STAT3 expression matching the upregulations of upstream miRNAs 29a and 22 (**Figures 4**, **5**). Although miR-22 has been identified as an *in vivo* protective factor in LPS-induced acute lung injury, research into the biomolecular functioning of miR-22 in relation to lung injury, and even more so in relation to liver injury, is scarce (34, 35).

Lastly, miR-215 showed minor upregulations in the ETC group at all timepoints, except at 24 h after trauma. Notably, this miRNA showed strong downregulations in the DCO group, most evidently at 24 and 72 h after trauma. One of its targets is CTNNBIP1, a protein that negatively regulates Wnt-signalling by binding to β-catenin, the main activator of the Wnt-signalling pathway (36). The expression of CTNNBIP1 was significantly downregulated in lung tissue from the ETC group as compared to that of the DCO group. This corresponds to the strong downregulation of miR-215 in the DCO group, reducing the post-transcriptional silencing of its target CTNNBIP1. Although miR-215 has been broadly investigated in various fields, suggesting for example a relation between lung cancer and the upregulation of miR-215, research on miR-215 in relation to trauma is lacking (37). The marked systemic downregulations upon less invasive surgery as compared to upregulations upon more invasive surgery do imply that there might be a systemic involvement of miR-215 after multiple trauma.

The nature of multiple trauma implies an abrupt alteration in physiology that changes the tissue’s overall state and functioning. Immune and tissue regenerative processes are subsequently activated, often starting with inflammation followed by more deliberate, intricate processes that aim at restoring tissues. EVs have gained interest in the field of trauma research due to their role as transporters of cellular mediators in these processes after multiple trauma (7). Limitations of this study are the 72 h duration of the model, which is too short to observe long term complications. The 72 h duration also makes it unfit to investigate clinical outcome in the long term. However, this study is among the first to investigate the expression and role of circulating, EV-packed miRNAs in a multiple trauma model in which two different surgical treatment strategies were compared. This study also showed that, in this translational multiple trauma model, DCO results in a reduced expression of pro-inflammatory miRNAs in the systemic circulation.

## 5 Conclusion

Multiple trauma is an extraordinary condition that, unlike other diseases, rarely presents with identical symptoms in different patients. Multiple trauma management can therefore be challenging since several injuries in different tissue types and organs require tailored treatment plans. The systemic circulation plays an important role in multiple trauma, first and foremost since it carries oxygen and nutrients to the tissues at stake, but also due to the role that it plays in communication between tissues. This study revealed distinctive, treatment specific EV-packed miRNA expression patterns that correlated to mRNA expressions in target organs. Furthermore, this study demonstrates that EV-derived miRNA expression is a lively process, revealing dynamic miRNA expression kinetics throughout the acute phase until 72 h after multiple trauma. Various of the identified miRNAs are involved in (systemic) inflammatory and tissue regenerative processes which correlated to the key injuries from the multiple trauma model. These miRNA expression patterns exhibited a clear systemic response to the increased surgical trauma in the ETC group, suggesting that inflammatory responses from the sustained injuries may be aggravated, and that the subsequent increase in systemic inflammation requires a more elaborate compensatory anti-inflammatory response. This study demonstrated that EV-transported miRNAs have a clear systemic involvement in multiple trauma, opening doors for further research into tissue-tissue talk in multiple trauma as well as therapeutic applications of EVs.

## Data availability statement

The original contributions presented in the study are publicly available. This data can be found here: https://doi.org/10.34894/NYKLDA.

## Ethics statement

The animal study was approved by the German governmental office of animal care and use LANUV (Landesamt für Natur, Umwelt und Verbraucherschutz Nordrhein-Westfalen, Recklinghausen, Germany) under the permit number 81.02.04.2020.A215. The study was conducted in accordance with the local legislation and institutional requirements.

## Author contributions

RG: Data curation, Formal Analysis, Investigation, Writing – original draft, Writing – review & editing. JG: Investigation, Writing – review & editing. ÜM: Investigation, Writing – review & editing. KH: Writing – review & editing. QZ: Investigation, Writing – review & editing. TB: Funding acquisition, Writing – review & editing. MH-L: Conceptualization, Funding acquisition, Writing – review & editing. FH: Conceptualization, Funding acquisition, Writing – review & editing. MG: Conceptualization, Data curation, Formal Analysis, Funding acquisition, Writing – original draft, Writing – review & editing.
