# Supplementary material for: Circulating miRNA expression in extracellular vesicles is associated with specific injuries after multiple trauma and surgical invasiveness
Source: Front Immunol. 2023 Oct 23;14:1273612. doi: 10.3389/fimmu.2023.1273612 (PMC10626999; doi:10.3389/fimmu.2023.1273612)
Supplement: Supplementary file 1 [file Table_1.docx]

Supplementary Material

**Supplementary Table 1.** Quantitative PCR primers: A Disintegrin and Metalloproteinase domain-containing protein 10 (ADAM10), Collagen Type 1 Alpha 1 Chain (COL1A1), Catenin Beta Interacting Protein 1 (CTNNBIP1), and Signal Transducers and Activators of Transcription 3 (STAT3).

| ***Gene*** | ***Forward primer 5’🡪 3’*** | ***Reverse primer 5’ 🡪 3’*** | ***Accession code*** |
| --- | --- | --- | --- |
| **ADAM10** | CACGAGAAGCTGTGATTGCC | CGGAGAAGTCTGTGGTCTGG | AF009615.1 |
| **COL1A1** | GGCTCCTGCTCCTCTTAGCG | CATGGTACCTGAGGCCGTTC | NM_000088.4 |
| **CTNNBIP1** | CTTCCTACTTCTGCCCAGCC | CAGGCAAACAGGTGCTCAAC | NM_020248.3 |
| **STAT3** | AGCAGTTTCTTCAGAGCAGGT | CACAATCCGGGCAATCTCCA | NM_139276.3 |

**Supplementary Table 2.** Number of miRNAs that resulted up or downregulated per treatment group and timepoint.

| ***Treatment group*** | ***Time point (h)*** | ***#Downregulated miRNAs*** | ***#Upregulated miRNAs*** |
| --- | --- | --- | --- |
| ***ETC*** | 1.5 | 4 | 40 |
|  | 2.5 | 6 | 51 |
|  | 24 | 12 | 12 |
|  | 72 | 7 | 22 |
| ***DCO*** | 1.5 | 17 | 13 |
|  | 2.5 | 7 | 42 |
|  | 24 | 27 | 9 |
|  | 72 | 35 | 5 |
